# Neighborhoods’ Evaluation: Influence on Well-Being Variables

**DOI:** 10.3389/fpsyg.2019.01736

**Published:** 2019-07-31

**Authors:** Cristina Ruiz, Estefanía Hernández-Fernaud, Gladys Rolo-González, Bernardo Hernández

**Affiliations:** Departamento de Psicología Cognitiva, Social y Organizacional, Facultad de Psicología, Universidad de La Laguna, Santa Cruz de Tenerife, Spain

**Keywords:** neighbourhood resources, residents’ perceptions of neighbourhood, well-being, residential satisfaction, reason for living in a place

## Abstract

The influence of neighborhood characteristics on residents’ well-being and residential satisfaction has been widely studied, and has presented considerable variability. This study analyses the extent to which neighborhood resources influence variables relating to well-being, and examines the relationship between neighborhood resources and residents’ perceptions. The study was structured over two phases: (1) the neighborhood resources were evaluated, and (2) 252 neighborhood residents was interviewed. The results have shown that the observation by independent observers of neighborhood resources is connected to residents’ perceptions of their neighborhood. Residents’ perceptions of their neighborhoods is associated with indicators of well-being, and residential satisfaction. Also, the reasons for living in the neighborhood appear to be connected to the observed availability of resources and the perception of it. Wellbeing and residential satisfaction are the outcome of multiple aspects that are not limited to structural and material elements of neighborhoods.

## Introduction

The influence of neighborhood characteristics on the experiences deriving from people’s interaction with their place of residence and, in particular, on residents’ health, well-being and satisfaction with their neighborhoods, has been widely studied in environmental psychology (Hur and Morrow-Jones, 2008; Oktay et al., 2012; Bonaiuto et al., 2015; Wheaton et al., 2015; Pasca et al., 2016; Lekkas et al., 2017). In order to explain the influence of neighborhood attributes, the authors refer to the role played by the objectively defined characteristics, residents’ perceptions, and the distinction between the objective and perceived characteristics of the environment that influence the positive or negative assessment made by residents.

As pointed out by Godhwani et al. (2019) it is not common to find studies that jointly take into account the objective and subjective evaluation of the neighborhood, and even less frequent to find studies that appraise how objective and subjective evaluations affect one another, and how they affect health over time.

Orstad et al. (2017) carried out a systematic review of studies on the relation between objective and perceived characteristics of residential neighborhoods. The results obtained by these authors show that the objective and subjective measurements of environment evaluation can be less comparable than their definitions suggest *a priori*. That is, the relation between the subjective evaluation of certain characteristics of the environment tends to reveal a low association with the objective measurements of these same attributes. Orstad et al. (2017) suggest that subjective and objective measurements of neighborhood attributes should not be used indistinctly because they may be drawing on various sources of variability in residents’ responses. It would be interesting to identify the objective variables of neighborhood assessment that can be related to subjective evaluation.

Moreover, Nickelson et al. (2013) point out that numerous instruments have been developed to evaluate neighborhood characteristics, the majority of which focus on responding to specific objectives of analysis. For example, some of them assess both the social and physical environment, while others only address the physical. These authors analyze the instruments created to assess the qualities of the residential environment with specific populations in concrete aspects of health, well-being and behavior, and identify up to 20 domains and around 300 subdomains used to classify the physical and environmental factors of residential environments. Examples of these domains and subdomains are: (a) the existence of services in outdoor areas (which comprise subdomains such as benches and/or covered shelters at public transport stops, public baths, urban furniture, etc.); (b) architectural characteristics (with subdomains: height and shape of construction, buildings with windows looking onto the street, among others); and (c) land use (e.g., plots/agricultural land, use for commercial, educational, industrial and production purposes, institutional buildings, etc.).

Research results that attempt to analyze the influence of neighborhood characteristics have also presented considerable variability. For example, by comparing the neighborhoods of two US regions based on the responses of 1,726 residents, Lee et al. (2017) found that people report higher satisfaction when they perceive certain neighborhood characteristics: (a) greater road safety for pedestrians and vehicles; (b) fast and varied access to destinations; (c) safety from delinquency; (d) lower residential density; (e) better access to outdoor recreation areas; and (f) attractive appearance. It is important to point out, however, that the coherence found between the neighborhood characteristics that influence satisfaction is only partial. Thus, Lee et al. (2017) indicate that the relation of residential satisfaction with lower room density and faster and more varied access to destinations is striking because, from a planning perspective, it is not possible to improve the accessibility and diversity of destinations without increasing residential density. Moreover, although the study uses both objective and subjective measurements of neighborhood characteristics, only the latter offer significant discriminative capacity.

On the same lines, a study by Wilkerson et al. (2012) concludes that the characteristics of the built environment play a vital role in the development of positive relations between neighbors. In an analysis of eight neighborhoods in Portland, Oregon (United States), objective and subjective data were gathered using various methodologies: (a) systematic observation through an audit that evaluated the physical characteristics of the streets in each neighborhood; (b) a survey of residents undertaken by trained interviewers who, in addition to gathering information on sociodemographic characteristics such as age, sex or race, included questions about the length of time living in the neighborhood, the perception of safety or self-evaluation of health, among other aspects relating to quality of life; and (c) objective data obtained from the land registry relating to the type of property of the dwellings in each neighborhood, their market value and age of construction. The results obtained suggest that knowledge, contact and trust between neighbors increase with physical and environmental characteristics that provide a semi-private space that enhances informal social interactions, such as front porches or balconies, and continuous pavements that facilitate transfer on foot from one place to another within the neighborhood and subsequently interaction with other people. In contrast, bars on windows and doors, graffiti and rubbish in the streets, objective characteristics of the environment associated with the perception of lack of safety, do not affect the positive bonds between neighbors in the neighborhoods analyzed. The authors conclude that macro-level physical and environmental characteristics are associated with residents’ sense of community.

These results may be indicating that people who live in different types of neighborhoods can take into account various aspects when determining their levels of satisfaction. In the study by Hur and Morrow-Jones (2008) on satisfaction with neighborhoods in Franklin, Ohio (United States), residents from neighborhoods evaluated as satisfactory and unsatisfactory emphasized the physical characteristics of the neighborhood when indicating their degree of satisfaction. However, residents from neighborhoods that scored as unsatisfactory concentrated more on the social characteristics associated with neighborhood problems when indicating their degree of satisfaction. These authors claim that there is no specific set of characteristics nor any standard rule to establish the degrees of satisfaction that can be applied to all areas of a city. In fact, other authors argue that residential satisfaction may depend too on emotional bonds with the place (Fleury-Bahi et al., 2008).

Conversely, the data obtained by Ibrahim Abass and Tucker (2018) in a study developed in three suburbs in the southwest of Geelong, Australia, show that neighborhood characteristics such as street type, tree coverage, and provision of sidewalks, shared open space and community spaces were found to be the most important predictors of neighborhood satisfaction. In this sense, places chosen for their physical characteristics, opportunities, services and facilities, which permit people establish instrumental and practical relationships with their area, are likely to produce a greater attachment, but too those that imply family connection or rootness, increasing behaviors that involve approximation to those places and desire to stay in them (Clark et al., 2017). It seems, therefore, that the reasons that lead a person to live in a neighborhood can influence the satisfaction with it.

In another study, Oktay et al. (2012) analyzed satisfaction and the perceived quality of the neighborhood, by comparing the responses of long-term and temporary residents in a city in Cyprus. Satisfaction with the neighborhood was measured generally and in relation to a set of specific physical and social attributes. The attributes that helped explain the satisfaction of habitual neighborhood residents were that it was a suitable place to live and level of noise. Conversely, the degree of satisfaction of temporary residents was linked to the attractiveness of the neighborhood, accessibility and upkeep of the physical environment. Oktay et al. (2012) understand that affective and social bonds are key to explaining satisfaction and the positive assessment of the quality of the neighborhood by local residents. However, in the evaluation of the quality of the neighborhood made by temporary residents, the physical attributes of the environment are more important than the social elements, in terms of measuring residential satisfaction.

Following a similar logic, Batson and Monnat (2015) claim that evaluating satisfaction with neighborhood is a solid measure of the physical and social qualities that may be visible to residents and visitors alike. However, satisfaction cannot be considered the single most important valorative bond to neighborhood. These authors, for example, sustain that the construct of residential quality of life refers equally to a more global psychological-emotional connection which is characteristic of neighborhood residents and is often beyond the grasp of people who do not live there. In their opinion, the concepts of satisfaction with neighborhood and residents’ quality of life refer to or measure different affective links with the environment. From this perspective, neighbors’ satisfaction with their area reflects the complex affective evaluations that residents make about how well their neighborhood satisfies their physical and social needs. Conversely, quality of life refers to more holistic experiences of well-being and not so much to the assessment of real conditions in the neighborhood. In this sense, according to Batson and Monnat (2015), neighborhood quality of life would be conceptualized as the set of characteristics of the conditions of life that enable residents to feel good and keep their physical, mental and social independence. In this way, the concept of residential quality of life encompasses the affective and cognitive components that people use to lend meaning and coherence to their own lives in relation to their residential surroundings. Several studies have found positive relation between quality of life, optimism and satisfaction with life. The interest of the optimism and life satisfaction in the studies on perception of the neighborhood lies in the fact that they can be related to processes of adaptation to the environment (Wrosch and Scheier, 2003).

Despite the research effort made, as pointed out by Parsons et al. (2010): “the nature of the relationship between neighborhood conditions and residents’ health (and the mediator and moderator factors at play) remains” (pp. 1). This study analyses the extent to which neighborhood resources, as observable neighborhood characteristic, influence variables relating to quality of life, such as social support, satisfaction with life, optimism and residential satisfaction. It also examines the relationship between neighborhood resources and residents’ perceptions. In order to reach these objectives, we first decided to develop instruments that would enable the objective evaluation of certain neighborhood characteristics and residents’ perceptions of them. The approach of the studies outlined above indicates that the reasons why residents live in their neighborhoods can alter the evaluations they make and influence the variables of satisfaction and quality of life. In this study, we inserted these variables in the analyses made, in order to provide an integrating image of how the neighborhood resources, the perception of residents and their own motivations could affect some of the measurements relating to quality of residential life.

## Materials and Methods

### Participants

The sample was composed of 252 residents in five different neighborhoods of the island of Tenerife (Spain). Men accounted for 48.4% and women for 51.6%. The average age was 47.3 years (*SD* = 20.3; range 18–89). Of them, 35.7% lived in a detached house, 63.5% in a flat or apartment, and 0.8% in guesthouses or private lodgings. Of the total sample, 66.7% owned their own home. The average time as residents in the neighborhood of the total sample was 22.10 years (*SD* = 18.5; range 0–85).

### Design

We used an *ex post* facto simple prospective design (Montero and León, 2007). The classificatory independent variables were Residential neighborhood (five neighborhoods with different levels of resources); Reasons for living in the neighborhood (four levels: family, economic, proximity, or environment); and Residents’ level of perception of the neighborhood [three levels (percentile groups): high, medium, low; high group means more positive perception of the neighborhood]. The dependent variables were Social support, Satisfaction with life, Optimism, Neighborhood perception and Residential satisfaction.

### Instruments

Two questionnaires were used: Observation of neighborhood characteristics and Residents’ evaluation questionnaire. Below is an outline of each one.

#### Observation of Neighborhood Characteristics Questionnaire

For this study, only the resources and services dimension of questionnaire developed by Ruiz et al. (2015) was used (presence of educational, social and health, commercial financial, recreational, and leisure resources). The observer was required to indicate the amount of resources and services in the neighborhood.

#### Residents’ Evaluation Questionnaire

In order to evaluate residents’ perceptions of neighborhoods, we created a questionnaire with the following scales:

(1) Quality of life scale (ComQol-S5): questions were taken from the first and second sections of the scale devised by Cummins (1997). Specifically, the variables chosen were Social support, and Satisfaction with life. Social support was calculated using three items referring to the frequency with which friends or family members were available for various activities (α = 0.67). Satisfaction with life was calculated through the linear combination of the final scale of nine items of the ComQol-S5 (α = 0.75).
(2) Life Orientation Test Revised (LOT-R): this scale is a revised form of Scheier et al. (1994) original scale to evaluate dispositional optimism. The psychometric properties of the scale in the Spanish version were tested by Ferrando et al. (2002). The scale consists of 10 items that use a five-point Likert response scale, where zero indicates total disagreement and four, total agreement. Of the total items, six measure the dimension of dispositional optimism and the remaining four are fillers. The internal consistency of the scale with this sample was 0.75.
(3) Perception of the neighborhood: based on the dimensions included in the neighborhood observation questionnaire (Ruiz et al., 2015), we created a scale of 23 questions where, with a range of response from 1 to 10, residents were required to evaluate the existence of various characteristics in their neighborhood. Internal consistency was 0.84.
(4) Residential satisfaction: at the end of the questionnaire, four questions were added about whether the neighborhood was considered recommendable, safe or with an appropriate standard of living, and were used to obtain a measurement of Residential satisfaction (α = 0.85).
(5) Reasons for living in the neighborhood: the objective of the last question was to classify residents according to their reasons for living in the neighborhood. Four alternative responses were offered: for family reasons, for the environment, for economic reasons (the economic level does not allow you to live in another neighborhood) and for accessibility to other places.

### Procedure

This study has been approved by the University of La Laguna Ethics Committee in Tenerife (Spain). This study was structured over two phases. In the first phase, the resources and services of the neighborhoods were evaluated by Observation of neighborhood characteristics questionnaire. Five observers were trained to evaluate the neighborhoods selected according to recommendations by Zenk et al. (2007). Five neighborhoods were assessed of the metropolitan area of the island. The mean population varies between the 4,159 inhabitants and the 6,147 inhabitants. Each neighborhood was assessed by three observers randomly distributed. Each neighborhood was to be visited on three different occasions and different routes were to be followed. Interjudge reliability analysis was based on the intraclass correlation coefficient. The interjudge concordance was high and significant for the resources dimension, obtaining values between 0.81 and 0.93. According to the level of resources, Neighborhood 1 (*M* = 331.33, *SD* = 88.48) and Neighborhood 2 (*M* = 321.66, *SD* = 73.92) stand out above the rest, and Neighborhood 5 (*M* = 183.00, *SD* = 16.46) and Neighborhood 4 (*M* = 217.33, *SD* = 28.74) obtains the lowest level. Neighborhood 3 (*M* = 261.66, *SD* = 37.63) show an intermediate level of resources.

In the second phase a sample of neighborhood residents was interviewed. The objective was to evaluate residents’ perception of neighborhood characteristics, residential satisfaction and the variables relating to quality of life. Residents of all five neighborhoods were interviewed. The sample was collected according to a system of quotas per age range and gender. The interviewers were five psychology graduates who were trained to apply the Residents’ evaluation questionnaire. The interviewers requested the participation of people who were either on the streets of the various neighborhoods or waiting in a public place (e.g., health center, residents’ association), having previously confirmed that they were resident in the neighborhood. If people agreed to participate the interviewer asked questions from the questionnaire, recording the responses on a laptop or tablet (Excel sheet). The average duration of the interview was 45 min. Interviews were made in October and November 2015.

### Ethics Statement

Because the study involved no risk to participants, informed consent was given verbally. Participants were clearly informed that the participation was voluntary and that there would be no compensation for participation. The University of La Laguna Ethics Committee in Tenerife, Spain (ULLECT) approved this study. All relevant data are available via the Harvard Dataverse at^1^.

## Results

One aim of this work was to check the level of coincidence between the level of resources and resident’s neighborhood evaluation, by contrasting observers’ neighborhood assessments and residents’ perceptions. Other objective was to check the effect of the physical characteristics of the neighborhood on the measurements of residential quality of life. The results are displayed in the following two sections.

### Level of Resources and Resident’s Evaluation of Neighborhoods

First of all, the Neighborhood perception scale was used to calculate an overall score of the level of positive perception of the neighborhood. This score was used to create a classification variable with three levels: low, medium and high. This variable was named Levels of perception.

An exploratory factor analysis with varimax rotation was used to check how the 23 items of the questionnaire were grouped. The solution obtained grouped the items in seven factors that explained 65% of the variance. These seven factors were named Environment (architecture, maintenance and upkeep), Green (green areas), Resources_P (services perceived to be on offer in the neighborhood), Disturbances (includes negative items of environmental health associated with noise or stressful variables), Relations (referring to the social dimension), Leisure (leisure and sports services), and Environmental health (relating to the positive variables of environmental health).

**FIGURE 1:**
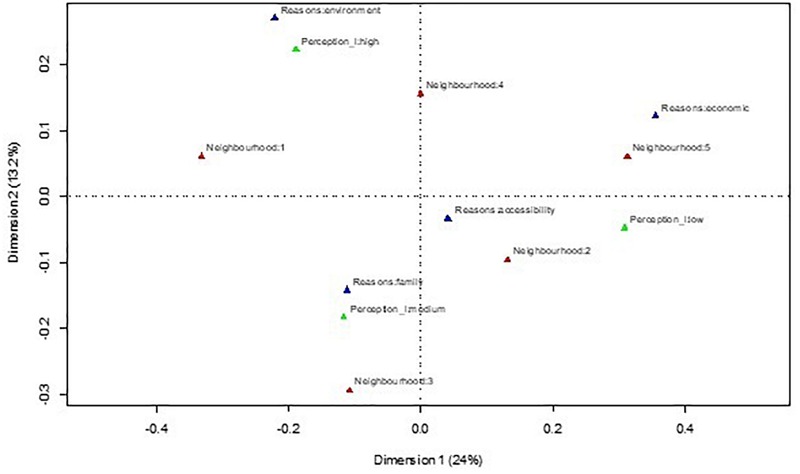
Correspondence analysis between neighborhood, levels of perception and reasons to live in the neighborhood.

A correspondence analysis was then made to check the association between the classification of neighborhoods by resources and the subjective perception of them, and a MANOVA was used to check the separation of neighborhoods, according to the seven factors obtained. The correspondence analysis was made using the classificatory variables Neighborhood, Levels of perception and Reasons for living in the neighborhood. The correspondence analysis constructs a cartesian diagram based on the association between the variables analyzed, where the proximity between the points represented is related to the level of association between the variables (Salvador, 2003). The neighborhoods can then be placed in a bidimensional space in relation to the other two factors: Reasons for living in the neighborhood and Level of residents’ subjective perception of the neighborhood (Figure 1).

Figure 1 shows that the neighborhood with the least resources (Neighborhood 5), according to the objective classification of the observers, was defined by a low level of perception, and the reasons for living there were economic. Neighborhood 3, which is a neighborhood with an intermediate level of resources, according to the observers, was equally represented in the correspondence analysis by an intermediate level of perception, and the grounds for living there were essentially for family reasons. The neighborhood categorized by observers as having most resources (Neighborhood 1) was represented in this diagram by a high level of perception and the reason for living there was because of the environment. Therefore, the amount of resources offered by the neighborhood coincides with perceptions of it.

The MANOVA was made with the seven factors obtained from the Perception scale as dependent variables and Neighborhood as a between-subjects factor. Table 1 shows the means and standard deviations of the factor scores for each neighborhood in each factor.

**TABLE 1:** Mean scores and typical deviations of the factorial scores by neighborhood in each factor.

	**Dimensions**													
	**Environment**		**Green**		**Resources_P**		**Disturbances**		**Relations**		**Leisure**		**E-health**	
	M	SD	M	SD	M	SD	M	SD	M	SD	M	SD	M	SD
Neighborhood 1	0.24	0.74	0.33	1.00	0.19	0.86	0.28	0.80	0.01	0.80	0.04	0.91	0.23	0.96
Neighborhood 2	0.10	0.90	–0.14	0.99	0.07	0.94	0.74	1.03	0.29	0.96	–0.21	1.10	0.00	0.96
Neighborhood 3	0.02	1.31	–0.29	0.94	0.053	0.95	0.29	0.89	0.12	0.91	–0.28	1.33	0.15	1.01
Neighborhood 4	–0.11	0.94	0.20	0.74	0.08	1.28	0.02	0.98	–0.18	1.21	0.18	0.76	–0.12	1.02
Neighborhood 5	0.09	0.76	–0.42	1.09	–0.28	0.86	–0.23	0.66	–0.29	0.98	0.20	0.89	–0.16	0.98

**FIGURE 2:**
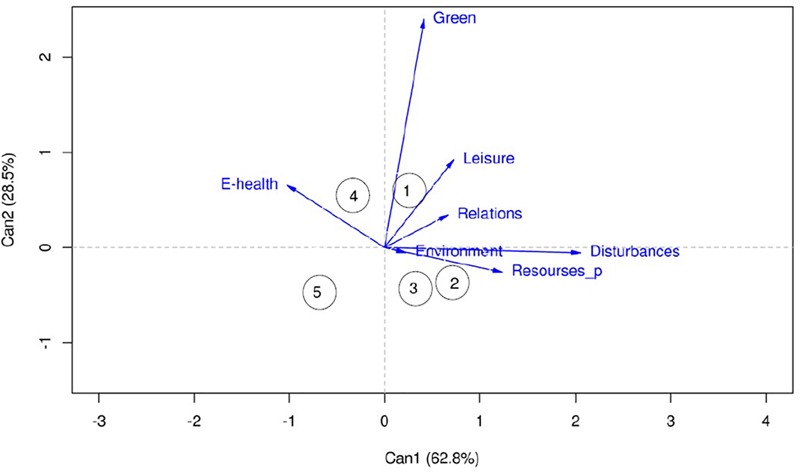
Canonical functions of the MANOVA for Perception Factors^*^Neighborhood.

The results showed a significant effect for Neighborhood [*F*(4, 28) = 3.54, *p <* 0.001] and two significant canonical functions, although the first explained the higher percentage of variance (62.8%). As shown in Figure 2, the first function separated the neighborhoods with fewer observed resources (Neighborhood 5 and Neighborhood 4) from the other three neighborhoods.

Table 2 gives the typical and structure coefficients for each variable and in this case both coincide positively for Disturbances, Resources_P, Leisure and Relations, and negatively for Environmental health, since living in a neighborhood with more resources can also include the perception of more disturbances and the loss of some positive sensations. The second function, which was essentially characterized by the weight of factors Green and Leisure, separated Neighborhood 1 and Neighborhood 4 from Neighborhood 5, Neighborhood 3 and Neighborhood 2. Neighborhood 1 and Neighborhood 4 effectively offer more resources in this sense because of the amount of parks and sports facilities. This separation generally coincides with the classification depending on the amount of resources observed.

**TABLE 2:** Manova results on perception factors by neighborhood.

	**Can1**	**Can2**
**Means**		
Neighborhood 1	0.23	–0.44
Neighborhood 2	0.73	0.23
Neighborhood 3	0.30	0.28
Neighborhood 4	–0.32	–0.38
Neighborhood 5	–0.71	0.32
**Typical coefficients**		
Environment	0.16	0.14
Green	0.02	–0.92
Leisure	0.29	–0.34
Disturbances	0.76	0.12
Resources_P	0.46	0.12
E-Health	–0.37	–0.27
Relations	0.24	–0.12
**Structure coefficients**		
Environment	0.08	0.01
Green	0.15	–0.87
Leisure	0.26	–0.33
Disturbances	0.74	0.01
Resources_P	0.44	0.09
E-Health	–0.37	–0.23
Relations	0.24	–0.12

Based on the results shown until now, we can consider the level of resources a useful tool for classifying them: neighborhoods with fewer resources were perceived more negatively by residents. However, although there is a general coincidence between observed resources and residents’ subjective evaluation, it is also true that personal variables, such as the reasons why neighborhoods are chosen as places to live, can change the evaluation and residential satisfaction.

### Effect of Neighborhood Characteristics on Quality of Life, Optimism, and Residential Satisfaction

For the second objective, in order to check the effect that perceived neighborhood characteristics may have on the different variables relating to quality of life, optimism and residential satisfaction, we first made an analysis of the correlations between them all. The results are interesting (see Table 3). The factor Environment, for example, correlated negatively with Social support, and positively with Life satisfaction, Optimism, and Residential satisfaction. The factor Resources_P had a positive relation with Optimism and Residential satisfaction (one of the highest). The factor Leisure correlated positively with Satisfaction with life and Optimism. The factor Disturbances correlated positively with Social support, and negatively with Satisfaction with life and Residential satisfaction.

**TABLE 3:** Correlations between well-being variables, factors of neighborhood perception, and residential satisfaction.

	**(1)**	**(2)**	**(3)**	**(4)**	**(5)**	**(6)**	**(7)**	**(8)**	**(9)**	**(10)**
Social support (1)										
L_satisfaction (2)	0.15^∗∗^									
Optimism (3)	0.13^*^	0.41^∗∗∗^								
R_satisfaction (4)	–0.09	0.27^∗∗∗^	0.22^∗∗∗^							
Environment (5)	–0.11	0.21^∗∗∗^	0.20^∗∗^	0.41^∗∗∗^						
Green (6)	0.14^∗∗^	0.00	–0.09	0.12	0.09					
Leisure (7)	0.08	0.15^*^	0.15^*^	0.24^∗∗∗^	0.04	0.01				
Disturbances (8)	0.18^∗∗^	–0.26^∗∗^	–0.12	–0.17^∗∗^	–0.15	0.10	0.03			
Resources_P (9)	–0.03	0.09	0.22^∗∗∗^	0.38^∗∗∗^	0.01	0.04	–0.04	–0.01		
E-health (10)	0.03	0.07	0.14^*^	0.10	–0.03	–0.06	0.02	0.03	0.02	
Relations (11)	–0.04	0.04	0.10	0.05	0.05	0.02	–0.02	0.02	0.04	0.04

Three ANOVAs were undertaken using Neighborhood^*^Levels of perception, Neighborhood^*^Reasons, and Levels of perception^*^Reasons as between-subjects variables. An ANOVA could not be performed jointly with all three factors because there were not enough cases in all the conditions. Optimism, Life satisfaction, Social support, and Residential satisfaction were used as dependent variables. Some significant effects were observed for the contrast Neighborhood^*^Levels of perception and are addressed below.

For the variable Optimism the effects of Neighborhood [*F*(4, 237) = 2.78, *p* = 0.02, η^2^ = 0.04] and Levels of perception [*F*(2, 237) = 8.14, *p <* 0.001, η^2^ = 0.06] were significant. The subsequent differences show that residents of neighborhoods with low resources (Neighborhood 5) present a lower mean in optimism than residents of other neighborhoods (see Table 4). Likewise, people with a more positive perception of their neighborhood are more optimistic than those who have a medium or lower opinion of their neighborhood.

**TABLE 4:** Descriptive statistics of dependent variables according to residents’ perception of each neighborhood.

	Optimism (0–4)		L_satisfaction (0–10)		Social support (1–5)		R_satisfaction (0–10)	
	M	SD	M	SD	M	SD	M	SD
Neighborhood 1	2.68	0.62	6.85	1.41	4.15	0.81	7.37	2.01
High	2.82	0.50	7.38	1.42	4.17	0.71	8.42	1.22
Medium	2.61	0.65	6.60	0.94	4.14	0.81	7.53	1.55
Low	2.43	0.78	6.00	1.70	4.11	1.11	4.36	1.37
Neighborhood 2	2.67	0.54	6.80	1.13	4.43	4.43	7.65	1.90
High	2.98	0.61	7.28	1.43	4.48	0.34	9.72	0.44
Medium	2.73	0.54	6.59	1.02	4.45	0.75	7.72	1.22
Low	2.47	0.45	6.76	1.06	4.39	0.71	6.60	2.03
Neighborhood 3	2.81	0.56	7.26	0.78	4.51	0.63	8.09	1.63
High	2.91	0.76	7.64	0.80	4.43	0.61	8.50	1.46
Medium	2.84	0.50	7.12	0.70	4.42	0.69	8.04	1.55
Low	2.72	0.52	7.18	0.83	4.67	0.57	7.90	1.86
Neighborhood 4	2.74	0.55	7.12	1.26	4.15	0.84	7.20	2.34
High	2.93	0.57	7.58	1.20	3.90	0.75	9.13	0.94
Medium	2.53	0.36	6.77	1.34	4.20	1.11	6.05	2.22
Low	2.70	0.61	6.86	1.13	4.41	0.61	5.92	2.12
Neighborhood 5	2.45	0.66	6.89	1.17	4.06	4.06	7.57	1.78
High	2.87	0.64	6.65	0.75	4.56	0.63	8.83	1.04
Medium	2.09	0.67	7.95	1.01	3.87	1.01	7.87	1.86
Low	2.47	0.57	6.55	1.16	3.93	0.70	6.84	1.68

The effect of Levels of perception [*F*(2, 236) = 15.25, *p <* 0.001, η^2^ = 0.11] was significant for the variable Life satisfaction (see Table 4), and residents whose perception of their neighborhood was more positive revealed greater satisfaction with life.

The effect of Neighborhood [*F*(4, 234) = 3.40, *p* = 0.01, η^2^ = 0.05] was significant for the variable Social support (see Table 4). Residents in neighborhoods with high or intermediate resources stated that they received more social support (see Table 4). Interestingly, in this case the neighborhood with the highest level of support is Neighborhood 3, which could be because this neighborhood is chosen on the basis of family ties.

**FIGURE 3:**
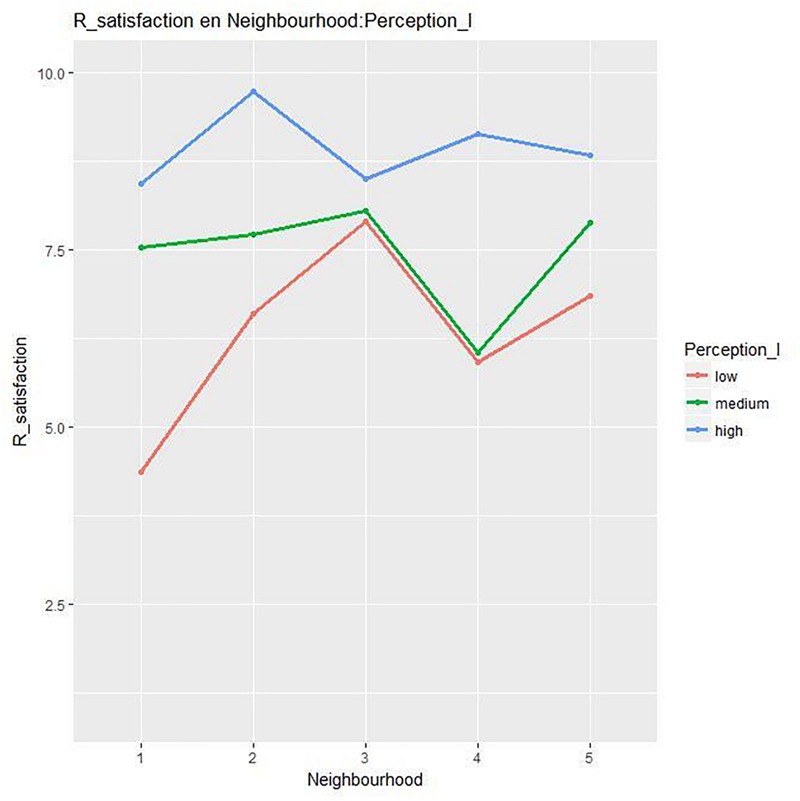
Differences in residential satisfaction by Neighborhood^*^Levels of Perception.

The interaction Neighborhood^*^Levels of Perception was significant [*F*(8, 237) = 3.77, *p <* 0.001, η^2^ = 0.11] for the variable Residential satisfaction. Table 4 shows the differences between high and low levels of perception in all the neighborhoods except Neighborhood 3. Neighborhood 3 was the neighborhood with the highest general residential satisfaction (see Figure 3).

**FIGURE 4:**
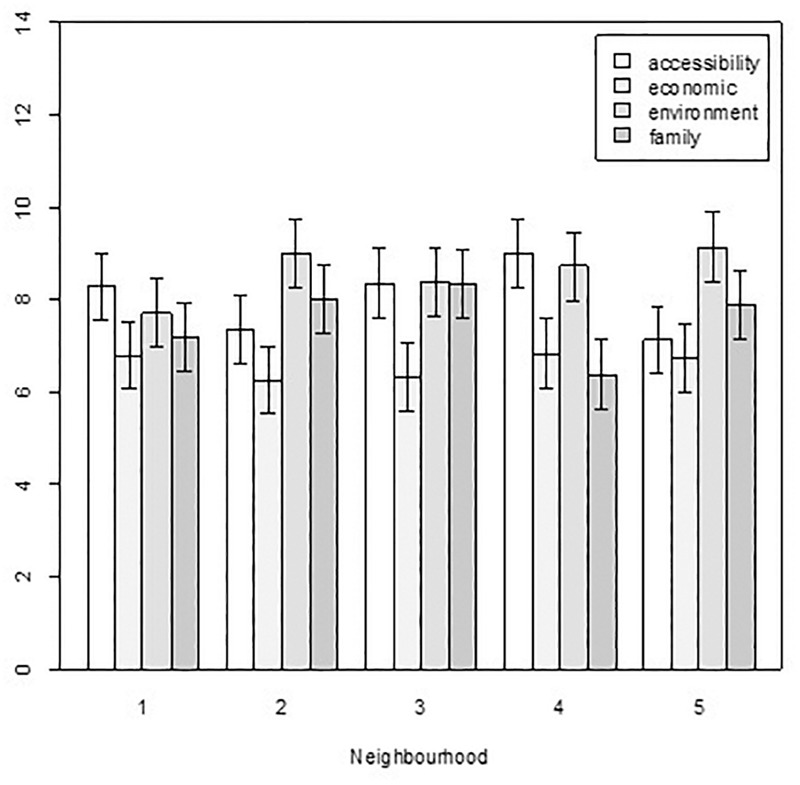
Differences in residential satisfaction by Neighborhood^*^Reasons.

The second ANOVA (Neighborhood^*^Reasons) only showed significant results for Residential satisfaction. There were significant differences for the factor Reasons [*F*(3, 224) = 9.22, *p <* 0.001, η^2^ = 0.10], and the mean was higher for residents who chose to live in their neighborhood because of the environment and lower for those who lived there for economic reasons (Means: environment: 8.670; family: 7.705; proximity: 7.688; economy: 6.627). Figure 4 shows that there were significant differences between the four types of reasons given in Neighborhood 5 and in Neighborhood 4. The degree of residential satisfaction was clearly higher in Neighborhood 5 when the neighborhood was chosen for the environment, while in Neighborhood 4 residential satisfaction was higher for those who chose the neighborhood for the environment but too for the proximity to points of interest.

**FIGURE 5:**
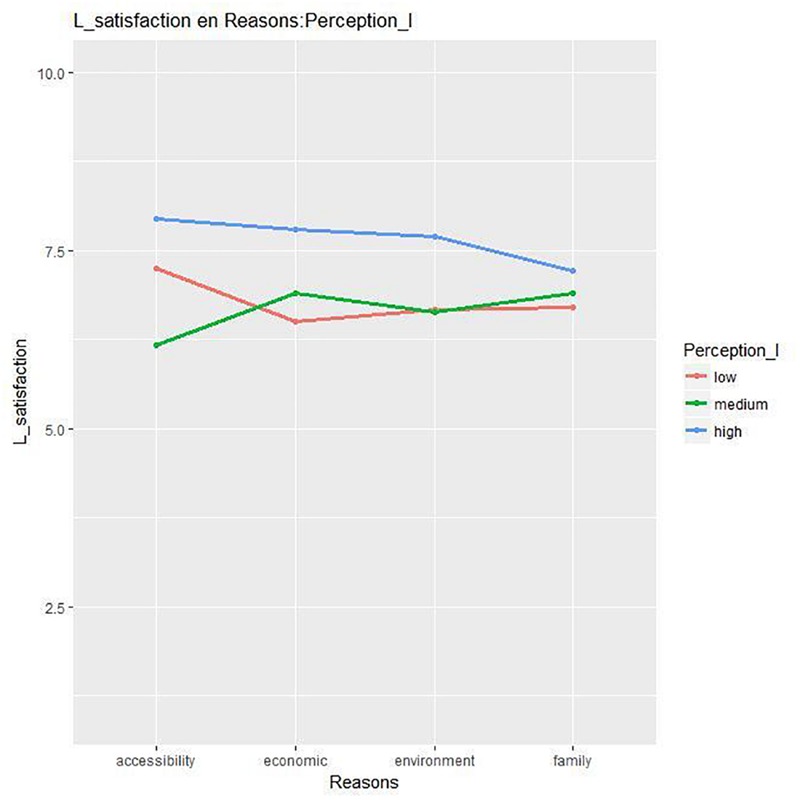
Differences in life satisfaction by Level of perception^*^Reasons.

The third ANOVA (Level of perception^*^Reasons) showed significant results for Life satisfaction. The interaction was significant [*F*(6, 231) = 2.17, *p* = 0.04, η^2^ = 0.05]. As show Figure 5, the mean in Life satisfaction is higher for people who perceive the neighborhood more positively except for those who chose the neighborhood for family reasons, for whom there is no difference between the three levels of perception in their life satisfaction. For the rest of the dependent variables, only the simple effects mentioned above were significant.

Both the correlations between neighborhood perception and the variables that reflect quality of life and mean differences found depending on the objective and subjective evaluation of neighborhood appear to indicate that environment is related to variables of psychological well-being.

## Discussion

This study contributes to furthering knowledge of the relationship between objectively defined neighborhood resources and residents’ perceptions of them, as well as the relationship between neighborhood characteristics and variables relating to quality of life. Specifically, this research is framed within studies that connect the impact of objective conditions of the urban environment to people’s perceptions and behaviors.

A methodological contribution of this study is to show that identification of neighborhood resources and services is a good indicator of its structural characteristics level, which would allow simplifying the procedures of evaluation of the environment. The results have shown that the observation by independent observers of services and resources available in a neighborhood is directly connected to residents’ perceptions of their own neighborhood, when social interaction, behaviors and, in general, processes that implies change, are not included in the observation, because those processes require specific observation measures (Valera et al., 2018). The lack of coincidence between objective and subjective measures found by Orstad et al. (2017) may be due to the fact that those changing characteristics of the analyzed spaces are included in the evaluation.

The results obtained are in line with the work that indicates that human beings are good evaluators of the conditions in which their lives develop. Specifically, in the studies comparing the self-evaluations carried out by patients with the subsequent clinical examination, a high percentage of coincidence is obtained, regardless of the self-evaluation procedure, the type of disease and the characteristics of the patients (Mazzotti et al., 2003). In the same way, objective and subjective estimates made in sports practice show a high degree of coincidence (Manzi et al., 2015).

Another result that should be highlighted is that residents’ perception of their neighborhoods is associated with indicators of well-being, quality of life in the neighborhood and residential satisfaction. Thus, residents with a more positive perception of their neighborhood showed greater residential satisfaction and satisfaction with life, or were more optimistic. Moreover, residents of the neighborhood with fewer resources revealed either a lower mean of optimism than residents in the other neighborhoods, or stated that they had less social support. The physical characteristics of the neighborhood seem to promote or maintain higher levels of optimism in its residents, though in this case it was in interaction with residents’ level of perception. Nevertheless, it is necessary to consider other alternative explanation in the sense that optimism as a dispositional characteristic can influence the positive or negative evaluation of the neighborhood, and it is not the perception of the neighborhood that modifies the optimism expressed. Even so, in line with Sanna et al. (2018), these results show the importance of subjective spatial characteristics for well-being.

It is also interesting to note that the reason why residents live in the neighborhood appears to be connected to the observed availability of resources and the perception of it. Thus, people who live in a neighborhood with few resources usually explain that they do so because their economic level do not allow them to live in another neighborhood and that they perceive their neighborhood as worse. However, those who live in neighborhoods with more resources usually agree that their choice was based on family ties or the environment, and that they have a better perception of them. The reason for which residents believe they remain in a neighborhood is undoubtedly an added source of satisfaction or dissatisfaction with it (Hui, 2013).

Similar results to those given here are found in studies that connect the quality of the neighborhood to residential satisfaction. In the study by Hur and Morrow-Jones (2008), residents of the neighborhoods analyzed underscored the physical characteristics of the neighborhood when indicating their degree of satisfaction. The results are also in line with those obtained by Lee et al. (2017), although these authors identify different variables of space from those used in this study. Oktay et al. (2012) also consider that the attributes that help explain the satisfaction of habitual neighborhood residents are twofold: the neighborhood is perceived to be a suitable place to live and the level of noise. Conversely, the degree of satisfaction of temporary residents was linked to the attractiveness of the neighborhood, accessibility and upkeep of the physical environment.

Also along the same lines are studies on the role of neighborhood characteristics on the influence on other personal variables, such as the establishment of attachment to place (Fleury-Bahi et al., 2008). Thus, Wilkerson et al. (2012) conclude that physical-environmental characteristics are associated with residents’ sense of community. The perception of physical care and danger in the neighborhood are variables that directly influence attachment to residential place (Tabernero et al., 2013). Therefore, the physical characteristics of the residential environment can influence the well-being and emotions that people experience on a daily basis. Not having any option but to live in an environment with worse conditions will have repercussions on residents’ quality of life.

From a psychological perspective, by revealing the interaction between reasons, perception, and real resources, once again the results indicate that residential satisfaction is the outcome of multiple aspects that are not limited to structural and material elements, but that require positive social interactions that can be enhanced by appropriate urban intervention. In this regard, observations show that although family and social ties could increase satisfaction, improved structural elements may also contribute.

In any case, the results must be taken with some caution, since the neighborhoods analyzed do not differ in other dimensions, such as the level of danger or confrontation between neighbors, which, according to other studies, could be of interest. This homogeneity between neighborhoods is connected to the type of neighborhood analyzed, since access to confrontational zones was avoided at all times.

## Data Availability

All datasets generated for this study are included in the manuscript and/or the supplementary files.

## Ethics Statement

The studies involving human participants were reviewed and approved by the University of La Laguna Ethics Committee in Tenerife, Spain (ULLECT). Written informed consent for participation was not required for this study in accordance with the national legislation and the institutional requirements.

## Author Contributions

CR conceived and designed the work, designed the instruments, acquired, analyzed, and interpreted the data, drafted the manuscript and revised it critically for important intellectual content, and approved the final version of the manuscript to be under-reviewed. EH-F conceived and designed the work, designed the instruments, acquired and interpreted the data, drafted the manuscript and revised it critically for important intellectual content, and approved the final version of the manuscript to be under-reviewed. GR-G conceived and designed the work, designed the instruments, drafted the manuscript and revised it critically for important intellectual content, and approved the final version of the manuscript to be under-reviewed. BH conceived and designed the work, designed the instruments, interpreted the data, drafted the manuscript and revised it critically for important intellectual content, and approved the final version of the manuscript to be under-reviewed.

## Conflict of Interest Statement

The authors declare that the research was conducted in the absence of any commercial or financial relationships that could be construed as a potential conflict of interest.

## Footnotes

1 https://dataverse.harvard.edu/dataset.xhtml?persistentId=doi:10.7910/DVN/XP9ZW2
